# Editorial: Patient and medical staff safety in the 21st century

**DOI:** 10.3389/fpubh.2022.1092149

**Published:** 2022-12-06

**Authors:** Wioletta Mędrzycka-Dąbrowska, Katarzyna Zorena, Adriano Friganović, Natalia Sak-Dankosky

**Affiliations:** ^1^Department of Anaesthesiology Nursing and Intensive Care, Faculty of Health Sciences, Medical University of Gdańsk, Gdańsk, Poland; ^2^Department of Immunobiology and Environment Microbiology, Faculty of Health Sciences, Medical University of Gdańsk, Gdańsk, Poland; ^3^Department of Nursing, University of Applied Health Sciences, Zagreb, Croatia; ^4^Department of Clinical Nursing, Medical University of Warsaw, Warsaw, Poland

**Keywords:** medical, safe, patient, WHO, environmental

Improving healthcare safety is a global priority and has been identified as an issue approaching epidemic proportions (1). The COVID-19 pandemic is a clear reminder of the importance of the safety of healthcare workers. Insufficient Personal Protective Equipment (PPE) has been a problem in many places, and there have been too many examples of infection and death of healthcare workers from COVID-19 (2). The number of adverse events that occur during the provision of medical services, and the associated costs are enormous. World Health Organization (WHO) reported that each year in middle-income countries there are ~134 million adverse events that result in failure to ensure safety in healthcare entities. As a consequence, 2.6 million people die annually (3).

Ensuring the safety of patients and medical staff is very difficult all over the world, regardless of the healthcare system model that is in place. The provision of medical services depends on the involvement of many representatives of various medical professions, methods of financing, condition of infrastructure, applied medical technologies, and the level of safety culture in the implementation of numerous processes, including nursing, diagnostic and therapeutic interventions. It is also important to consider how patient safety can be impaired when the health and safety conditions of health workers are not guaranteed. The interrelationship between occupational health and patient safety needs to be explored more to better understand how we can improve health services (4).

In this Research Topic, there are five manuscripts that address some of the problems related to the concept of safety in health care today.

Vuorio and Bor described an issue of the safety of healthcare staff in warzones, stating it is one of the most important yet often ignored humanitarian issues of the present. Currently, many medical staff working in Ukraine are experiencing severe stress. One of the consequences might be developing post-traumatic stress disorder (PTSD), a well-documented issue among deployed military healthcare workers (5). What is more, large numbers of refugees from the conflict zone arrive in different parts of the continent, which challenges almost all of Europe's healthcare systems. This also increases the possible burden of healthcare staff involved in taking care of them. However, those who directly deal with the war will suffer the most. The International Council of Nurses (ICN) emphasized that the safety of healthcare workers during this conflict is paramount (6).

Zhu et al. presented an observational study describing the problem of physicians' workflow interruptions in outpatient departments in China. They emphasized that unjustified interruptions of physicians' attention away from the current task might negatively impact the quality and efficiency of care (7). The most common reasons for those interruptions are: patients and their relatives, intra-departmental communication, and telephone/beeper calls (8–10) (Zhu et al.). They can disturb thought processes and increase cognitive demand, increasing the risk of errors and hampering patients' safety (Zhu et al.).

Heroor et al., with a multinational panel of experts, mapped out consensus statements for surgeons and operating room staff regarding practical management of surgical smoke safety, mitigating the risks associated with it. Surgical smoke generated by Energy devices used in OR might have a negative impact on all persons working in its environment. One of the most serious consequences includes mutagenic effects from the carcinogens present in the surgical smoke (Heroor et al.). As per an occupational safety and health administration (OSHA) study, ~500,000 healthcare workers including surgeons, nurses, anesthesiologists, and surgical technicians are exposed to surgical smoke every year (11). However, no substantive data on the extent to which recommendations regarding this issue have been implemented has been available. The consensus statement presented by the authors summarizes the common approaches and statements regarding preventing OR personnel from the hazardous effects of surgical smoke.

Wang et al. in their manuscript reflected on an issue of the mental health status of medical staff exposed to workplace violence. Hospital violence is one of the most commonly reported types of workplace violence and has a negative impact on staff's physical and mental health. Hospitals should implement different interventions in order to protect staff who experiences this issue (Wang et al.).

Finally, Shen et al. in their opinion manuscript presented an interesting solution for protecting the surgical team against COVID-19. They argued that using a concept of “Zero Contact,” which means a complete separation of uninfected personnel from infectious sources, to prevent the spread of infectious diseases could be a promising way to protect the staff. Specifically, they described a “Zero Contact” operation based on a robotic surgical system—the system originally designed for remote surgeries (12) but which could now be used instead to protect the staff from COVID infections (Shen et al.).

The above articles which reflect on safety issues can contribute to addressing the problems they describe and therefore improve staffs' wellbeing and thus the quality of care they provide.

## Author contributions

All authors listed have made a substantial, direct, and intellectual contribution to the work and approved it for publication.
